# Risk factors for premature coronary artery disease (PCAD) in adults: a systematic review protocol

**DOI:** 10.12688/f1000research.74926.1

**Published:** 2021-12-02

**Authors:** Adeel Khoja, Prabha H. Andraweera, Zohra S. Lassi, Mingyue Zheng, Maleesa M. Pathirana, Anna Ali, Emily Aldridge, Melanie R. Wittwer, Debajyoti D. Chaudhuri, Rosanna Tavella, Margaret A. Arstall

**Affiliations:** 1 Adelaide Medical School, Faculty of Health and Medical Sciences, University of Adelaide, Adelaide, South Australia, 5005, Australia; 2 The Robinson Research Institute, University of Adelaide, Adelaide, South Australia, 5006, Australia; 3 Department of Cardiology, Lyell McEwin Hospital, Elizabeth Vale, South Australia, 5112, Australia; 4 School of Health and Rehabilitation, Chengdu University of Traditional Chinese Medicine, Chengdu, Sichuan, China; 5 Department of Cardiology, Basil Hetzel Institute for Translational Health Research, The Queen Elizabeth Hospital, Woodville South, South Australia, 5011, Australia; 6 Cardiology Unit, Northern Adelaide Local Health Network, Adelaide, South Australia, 5112, Australia; 7 Medical Specialties, Faculty of Health Sciences, University of Adelaide, Adelaide, South Australia, 5005, Australia

**Keywords:** Coronary artery disease; premature coronary artery disease; risk factors; age; gender

## Abstract

PCAD possesses a public health challenge resulting in years of productive life lost and an escalating burden on health systems. Objective of this review is to compare modifiable and non-modifiable risk factors for PCAD compared to those without PCAD. This review will include all comparative observational studies conducted in adults aged >18 years with confirmed diagnosis of PCAD (on angiography) compared to those without PCAD. Databases to be searched include; PubMed, CINAHL, Embase, Web of Science, and grey literature (Google Scholar). All identified studies will be screened for title and abstract and full-text against the inclusion criteria on Covidence software. Data relevant to exposures and outcomes will be extracted from all included studies. All studies selected for data extraction will be critically appraised for methodological quality. Meta-analysis using random-effects model will be performed using Review Manager 5.3. Effect sizes for categorical risk factors will be expressed as odds ratios with 95% confidence intervals. For risk factors measured in continuous form, mean difference (if units are consistent) otherwise standardized mean difference (if units are different across studies) will be reported. Heterogeneity between

studies will be assessed using I ^2^ test statistics. GRADE will be used to assess the certainty of the findings.

*Systematic review registration number:* PROSPERO Registration # CRD42020173216

## Introduction

Coronary artery disease (CAD) is the most common type of heart disease and remains the leading cause of death worldwide, accounting for over 350,000 deaths each year. ^1^ The prevalence of CAD based on the global burden of disease (GBD) data is 154 million which translates into approximately one-third of the global burden of cardiovascular disease and 2% of the overall GBD. ^2^ Furthermore, in 2019, every fifth adult aged <65 years died from CAD. ^1^ The mortality of CAD over the last three decades in adults ≥65 years has declined to 5% among women and 4% among men. In contrast, over the last two decades, there has been a minor improvement of only 0.1% in terms of CAD mortality among adults <55 years of age. ^3^

CAD is defined as the reduction of blood flow to the heart muscle due to the build-up of plaque (atherosclerosis) in the arteries of the heart. ^4^ Premature coronary artery disease (PCAD) is defined as CAD occurring in men and women younger than 45 and 55 years respectively, but these cut-offs tend to vary from 45 to 65 years of age, as evident in different studies. ^5^^–^^8^ Early-onset CAD is used interchangeably with PCAD or often termed as CAD in young adults. ^9^ Late-onset CAD is defined as CAD experienced in men aged >55 years and in women aged >65 years. ^10^ Late-onset CAD is also used interchangeably with the normal occurrence of CAD. The prevalence of PCAD in a large survey done in German cardiac rehabilitation centres revealed 37% for men aged <55 years and women aged <65 years, while the prevalence for CAD was 67% for men aged >55 years and women aged >65 years. ^10^

Globally, PCAD represents a significant burden and an important public health challenge resulting in years of productive lives lost and an increased burden on health systems. More than four-fifths of those who present with PCAD have at least one modifiable risk factor. ^10^^,^^11^ Overall, the incidence of PCAD has minimally declined and of even more concern is that the prevalence in individuals <65 years with three major known risk factors (i.e. diabetes, hypertension, and obesity) has increased between 2000 to 2016. This translates into more risk burden and mortality rates in young adults experiencing PCAD. ^12^ The known modifiable risk factors for PCAD include, but are not limited to, smoking, high blood pressure, diabetes mellitus, physical inactivity, obesity, dyslipidaemia, and psychosocial stress. ^13^^,^^14^ Non-modifiable risk factors include, but are not limited to, age, gender, ethnicity, family history of heart disease, and homocystinuria. ^15^^,^^16^ The following three paragraphs will be talking about gender differences on the incidence of PCAD and comparing risk factors for PCAD compared to those without PCAD.

Gender differences in risk profiles have a substantial impact on the incidence of premature coronary artery disease based on earlier systematic reviews. ^17^^,^^18^ In general, women tend to be 10 years older than men at the time of the first episode of CAD. ^17^ Women also experience atypical symptoms of CAD such as fatigue, cramps in the abdomen, anxiety, nausea, and vomiting, and indigestion at the time of initial presentation. ^17^^,^^19^ Studies have reported a longer time duration from symptom onset to diagnosis among women with CAD. ^20^^,^^21^ In addition, the population attributable risk (PAR) of conventional risk factors for PCAD among men (aged ≤55 years) and women (aged ≤65 years) accounted for 93% and 97%, respectively. ^22^ Therefore, it is critical to identify differences in risk factors between men and women having PCAD.

Different patterns of risk factors for premature compared to late-onset CAD have been highlighted by an earlier systematic review and data from a population-based registry. ^10^^,^^23^ Conventional risk factors such as; smoking, family history of CAD, opium use, and dyslipidaemia are considered to be dominant in PCAD as compared to risk factors such as; hypertension, obesity, sedentary lifestyle and diabetes mellitus which are more prevalent among those who experience late-onset disease. ^6^^,^^10^^,^^23^^,^^24^

Different patterns of risk factors for patients with PCAD compared to healthy individuals having normal coronary arteries have been identified by a systematic review and a large-scaled survey done in Iran, which revealed that risk factors such as; dyslipidaemia (52%), cigarette smoking (66%), and strong family history of CAD (90%) individually accounted for increased risk of PCAD as compared to controls. ^15^^,^^25^^,^^26^ Since PCAD is a multifactorial disease and genetic predisposition plays an important role in younger adults diagnosed with this disease, it is imperative to further investigate risk factors between patients with PCAD compared to those without PCAD. ^27^

All major risk factors i.e. modifiable and non-modifiable have not been explored comprehensively in earlier systematic reviews targeting patients with PCAD. ^10^^,^^23^^,^^28^ They either included selected risk factors for PCAD, targeted certain sub-groups, or were restricted to a specific country. To date, no comprehensive appraisal of the evidence has been conducted; and a preliminary search of PROSPERO, PubMed, Google Scholar, and the Joanna Briggs Institute (JBI) Evidence Synthesis did not identify any ongoing systematic review on the current topic. The understanding of modifiable and non-modifiable risk factors is critical for the prevention of morbidity and mortality associated with PCAD. Although, there are a number of published observational studies on PCAD risk factors, yet a thorough systematic review of the evidence is lacking. The results of this systematic review will add to the existing scientific knowledge and will inform current clinical practice guidelines. The objective of this review is to compare the prevalence of modifiable and non-modifiable risk factors in patients with PCAD compared to those without PCAD.

### Review questions

The objective of this review is to compare the prevalence of modifiable and non-modifiable risk factors for PCAD compared to those without PCAD among the following groups:

1. Patients experiencing PCAD as compared to those experiencing late-onset CAD
2. Patients experiencing PCAD as compared to healthy controls

### Inclusion criteria

*Population*

This systematic review will consider studies that target participants of both sexes, aged ≥ 18 years with a confirmed diagnosis of PCAD on angiography. We will include studies that compare risk factors among patients with PCAD compared to those without a diagnosis of PCAD according to the two groups mentioned above.

*Exposure of interest*

Exposure would be risk factors (modifiable and non-modifiable). Modifiable risk factors will include (but not limited to); smoking, hypertension, diabetes mellitus, physical inactivity, obesity, high body mass index (BMI), high cholesterol, substance abuse; and the non-modifiable factors will include (but not limited to); gender, race/ethnicity, family history of heart disease, homocystinuria, polymorphism of MTHFR gene, lipoprotein a. For a detailed list of exposures (Supplementary file 1), which has been selected based on extensive literature search and clinical relevance. However, outside the usual factors reported above and in supplementary file 1, risk factors such as pollution and dust particles, dental hygiene and various infections associated with atherosclerotic plaque rupture, rheumatoid arthritis, and associated medications including non-steroidal anti-inflammatory drugs (NSAIDs), obstructive sleep apnoea, sleep hygiene, job strain and low social support will not be part of this review, since literature suggesting a stronger mechanistic association between unusual risk factors mentioned above and PCAD is lacking; and as a result, it will be difficult to perform the meta-analysis.

*Outcomes*

The outcome of interest are patients with a confirmed diagnosis of PCAD based on angiography findings, as compared to patients with no PCAD across two study comparison groups. For this review, an age cut-off of ≤65 years (upper-limit) for both men and women will be used to differentiate PCAD from late-onset CAD. For this systematic review, controls will be defined either as healthy individuals having no disease or having any other disease not related to PCAD or CAD.

*Types of studies*

All comparative observational studies including prospective and retrospective cohort studies, case-control studies, and analytical cross-sectional studies will be eligible. Randomized controlled trials (RCTs), reviews including narrative and systematic reviews, conference abstracts, case series, and case reports, editorials and commentaries will be excluded.

## Methods

This systematic review protocol will follow the checklist of Preferred Reporting Items for Systematic Review and Meta-Analysis Protocols (PRISMA-P) (Supplementary file 2). ^29^ This protocol will facilitate in conducting a systematic review and meta-analysis on modifiable and non-modifiable risk factors related to PCAD for the two review questions. Any deviations to the protocol while conducting this review will be reported in the method section of the final manuscript. The review protocol is registered in the International Prospective Register of Systematic Reviews (PROSPERO) CRD42020173216.

### Search strategy

The search strategy aims to identify both published and non-published articles. An initial comprehensive search strategy which has been designed for PubMed in consultation with the librarian specialized in scientific medical literature at the University of Adelaide, Australia including the MeSH (medical subject headings), will be later translated across different databases for the title and abstract search (Appendix I). In addition, the reference list of all the included studies and systematic reviews that have been excluded will be cross-checked to identify any relevant studies that might be included for the review. Since this review is a non-funded PhD thesis project we lack resources for translation services, hence studies that are published in English language only and available as full-text will be considered for inclusion in this review. A search range based on publication year will not be set to allow more studies to be part of this review.

### Information sources

The following databases will be searched from inception: PubMed (MEDLINE), CINAHL (EBSCOhost), Embase (Elsevier), and Web of Science (Clarivate). The search for non-published (grey literature) and ongoing studies will include Google Scholar ( https://scholar.google.com/), ClinicalTrials.gov ( https://clinicaltrials.gov/), American Heart Association ( https://www.heart.org/), American College of Cardiology ( https://www.acc.org/), the World Health Organisation ( https://www.who.int/), and the Centres for Disease Control and Prevention ( https://www.cdc.gov/).

### Study selection

All identified citations will be collated and exported to a reference management software EndNote X9 (Clarivate Analytics, PA, USA) and initial duplicates will be removed at this stage. The studies will then be imported to online systematic review software, Covidence ( https://www.covidence.org/). The second stage of duplicate removal will be performed on Covidence. Title and abstract screening will be undertaken independently by two reviewers (AK, MP) for assessment against the inclusion criteria of the review. The second stage will consider studies that meet the inclusion criteria and will be retrieved in full and articles will be uploaded on Covidence. The full-text of the selected studies will be assessed independently by two reviewers (EA, MW). Full-text articles that do not meet the eligibility criteria will be excluded and reasons for exclusion will be presented in the appendix section of the main systematic review. The results of the overall search and the studies included for meta-analysis will be presented as a PRISMA flow diagram (please refer to Figure 1). ^29^ Disagreement at any stage that arises between reviewers will be resolved through discussion or in consensus with a third reviewer (RT or MA).

### Assessment of methodological quality

All studies selected for data extraction will be critically appraised for methodological quality independently by two reviewers (ZSL, PA) using a standardized critical appraisal tool for comparative observational studies designed by National Heart, Lung, and Blood Institute (NHLBI) (Appendix II). ^30^ This quality assessment tool is designed to assist reviewers in focusing on concepts that are key for critical appraisal of the internal validity of a study and it is not designed to provide a list of factors comprising a numeric score. This tool is specific to individual types of included study designs. It includes items for evaluating potential flaws in study methods, including sources of bias (e.g., patient selection, performance, attrition, and detection), confounding, study power, the strength of causality in the association between exposures and outcomes, and other factors. When assessing quality, reviewers can select “yes,” “no,” or “cannot determine/not reported/not applicable” in response to each item on the tool. For each item where “no” is selected, reviewers are instructed to consider the potential risk of bias that can be introduced by that flaw in the study design. Cannot determine and not reported are also noted as representing potential flaws. Reviewers are instructed to rate this tool on the range of items included in each tool as mentioned above, to judge each study to be of “good”, “fair”, or “poor” quality. A “good” study has the least risk of bias, and results are considered to be valid. A “fair” study is susceptible to some bias deemed not sufficient to invalidate its results while a “poor” rating indicates a significant risk of bias.

Authors of the included studies will be contacted to obtain any missing information if needed. Disagreements that arise as a result of this appraisal will be resolved through discussion or in consensus with a third reviewer (RT or MA). The findings of this appraisal will be tabulated in a quantitative form and the studies will not be excluded based on the methodological quality. This assessment will in turn provide a thorough evaluation of the quality of evidence and the role of bias on the findings. For studies that are included more than once due to reporting of the data for at least one of the two review questions, the assessment of the methodological quality for each review question will be performed separately.

### Data extraction

Data will be extracted from the included studies using a structured data extraction form designed by the authors. Data extraction will be performed independently by two reviewers (MZ, AA).

The data extracted will include authors, publication year, study design, country of publication, time of assessment for PCAD, inclusion criteria for determination of cases, which will be defined as patients having PCAD, while controls will be defined as patients experiencing late-onset CAD or healthy controls (having no disease or without the disease of interest) (Appendix III, IV). To account for confounding, data on age, gender, and geographical settings will be extracted. All data will be extracted in alignment with the review questions. If a given study, fulfils the criteria of being included in more than one comparison group, the data extracted from that study will be analysed for each relevant comparison group. In addition, evaluation of duplicate published studies will be undertaken, the reviewers will identify the primary publication from the given set of studies from the same project. The primary study will be the one that has; the maximum sample size, maximum centres/countries involved, and maximum relevant outcomes reported.

Any disagreements that arise as a result of this appraisal will be resolved through discussion or in consensus with a third reviewer (RT, MA). Authors of the included studies will be contacted for any missing data if and when required.

### Data synthesis

The meta-analysis will be performed using RevMan software (Review Manager version 5.3) for each modifiable and non-modifiable risk factor. A random-effects model will be selected to account for heterogeneity in population across studies due to variation in genetic makeup including ethnic and racial differences. An overall pooled analysis of patients with PCAD compared to those without PCAD will be performed on crude estimates i.e. events, totals, mean and standard deviation from the included papers, stratified by age, gender, and geographical settings. For continuous variables, if the quantitative units vary across studies, Standard Mean Difference (SMD) and if the units are consistent then Mean Difference (MD) with respective 95% confidence intervals (Cls) will be reported. For dichotomous variables, odds ratio (OR) and 95% Cls will be reported.

Substantial heterogeneity between studies will be considered if the I ^2^ statistic exceed 30-50% and by visually inspecting the forest plots. If high levels of heterogeneity between studies are identified, further exploration of the data will be conducted by sub-group analysis. Sub-group analysis will be performed on age, gender, and geographical settings (high compared to low-income countries) for patients with PCAD compared to those without PCAD. In addition, sensitivity analysis will also be performed on studies that score “good” or “fair” after excluding studies that score “poor” on the NHLBI quality assessment tool to evaluate the effect on outcomes. To assess publication bias, funnel plots with 10 or more studies will be plotted to visualise all the included studies for meta-analysis. At instances, where statistical pooling is not possible, the findings will be represented in a narrative form in the tables and figures as deemed appropriate to aid in data presentation.

### Assessing certainty in the findings

To grade the certainty of evidence generated, this review will utilise the Grading of Recommendations Assessment, Development and Evaluation (GRADE) ^31^ approach and a Summary of Findings (SoF) table will be created using GRADEpro GDT (McMaster University, ON, Canada). The SoF table will include the following information where appropriate: absolute effects for all exposures (modifiable and non-modifiable risk factors), an estimate of relative effect, number of participants with associated studies and a ranking of the quality of evidence-based on the risk of bias, directness, heterogeneity, precision, and risk of publication bias of the review results. The outcomes to be reported in the SoF table will include PCAD, late-onset CAD and healthy controls (Appendix V).

## Discussion

To the best of our knowledge, this review will be the first to observe a comprehensive list of risk factors including modifiable and non-modifiable for PCAD across subpopulations. This review is anticipated to classify research gaps in the existing literature and provide evidence for further studies on risk factors for PCAD. Moreover, this review will also conduct sub-group analyses by comparing risk factors for PCAD between LMICs versus HICs (geographical settings).

We acknowledge the potential limitations as part of this systematic review. We will limit our search to articles published in English only, so might miss studies published in other languages. The other limitation is, that non-conventional risk factors such as; pollution and dust particles, dental hygiene and various infections associated with atherosclerotic plaque rupture, rheumatoid arthritis, and associated medications including non-steroidal anti-inflammatory drugs (NSAIDs), obstructive sleep apnoea, sleep hygiene, job strain and low social support will not be part of this review, since literature suggesting a stronger mechanistic association between the risk factors mentioned above and PCAD is lacking, as a result, it will be difficult to perform meta-analysis.

The findings of this review will support academics, researchers, healthcare professionals, relevant stakeholders and policy makers to focus on modifiable and non-modifiable risk factors for PCAD specific to age and gender and design appropriate preventive strategies as early as practical in order to significantly reduce the risk of PCAD and associated cardiovascular diseases later in life. The results of this systematic review will be publicly available and will be disseminated via academic presentations (both locally and internationally) and through peer-reviewed publications.

## Data availability

### Underlying data

Figshare: Risk factors for premature coronary artery disease (PCAD) in adults: a systematic review protocol, https://doi.org/10.6084/m9.figshare.17078507.v2. ^32^

Data are available under the terms of the Creative Commons Zero “No rights reserved” data waiver (CC0 1.0 Public domain dedication).

### Extended data

Figshare: Risk factors for premature coronary artery disease (PCAD) in adults: a systematic review protocol, https://doi.org/10.6084/m9.figshare.17078486.v2. ^33^

Data are available under the terms of the Creative Commons Zero “No rights reserved” data waiver (CC0 1.0 Public domain dedication).

## Authors contribution

AK, PHA, MAA, ZSL conceived and designed the study. AK drafted the manuscript, secured funding for this systematic review, and is the guarantor of the systematic review. AK, PHA, and ZSL developed the search strategy, research questions and study design. MZ, EA, MRW, MMP, AA, DDC, AK, PHA, ZSL designed the tables for data extraction, will perform data extraction, and also evaluate the quality of all included studies in the systematic review. PHA, ZSL, MZ contributed to the introduction section and supplementary files 1 and 2. AK, PHA, ZSL will contribute to data synthesis and meta-analysis. RT and MAA provided direction, mentorship and extensively revised the manuscript. All the authors reviewed and approved the final manuscript as submitted and agreed to be responsible for all aspects of the work.

## Funding

This systematic review protocol is part of a student based doctoral thesis project. Adeel Khoja is in his second year of doctorate, affiliated with Adelaide Medical School, The University of Adelaide and is currently on a fully-funded scholarship, “Adelaide Scholarship International” which covers all the cost associated with his thesis project and has no role in developing the review protocol.

## Study status

Currently, we have completed the screening of title and abstract for this comprehensive systematic review and have retrieved papers for full-text screening. After that, we will start data extraction of all those included full-text studies.

## Conflicts of interest

The authors declare no conflict of interest.
