# Supplementary material for: Risk factors for premature coronary artery disease (PCAD) in adults: a systematic review protocol
Source: F1000Res. 2021 Dec 2;10:1228. [Version 1] doi: 10.12688/f1000research.74926.1 (PMC8772521; doi:10.12688/f1000research.74926.1)
Supplement: Peer review history

# Peer review: Reviewer response for version 1

**DOI:** 10.5256/f1000research.78730.r101954

**Published:** 2022-01-19

**Authors:** Sobiya Sawani

**Affiliations:** 1 Department of Community Health Sciences, The Aga Khan University, Karachi, Pakistan

This seems a very interesting and comprehensive systematic review protocol targeting modifiable and non-modifiable risk factors for patients with premature coronary artery disease and comparing it to those without premature coronary artery disease. I strongly believe this is one of the rarest systematic review stratifying on age, gender and geographical settings (comparing high-income countries versus low-income countries). I would suggest the authors to make age categories as per the articles that form part of the meta-analysis (after data extraction), in order to explore risk factor profile in lieu to the defined age categories for premature coronary artery disease and this can be part of the sub-group analysis as you may not see more burden in the age bracket of 18-25 years; also the risk factors (modifiable and non-modifiable) may vary. In addition, this systematic review will be further strengthened by quality assessment and sensitivity analysis of studies that score good or fair on the quality assessment too, NHLBI after excluding studies that score poor. I also like the thought that four different databases will be included as part of this systematic review.

Is the study design appropriate for the research question?

Yes

Is the rationale for, and objectives of, the study clearly described?

Yes

Are sufficient details of the methods provided to allow replication by others?

Yes

Are the datasets clearly presented in a useable and accessible format?

Yes

Reviewer Expertise:

Communicable and non communicable diseases

I confirm that I have read this submission and believe that I have an appropriate level of expertise to confirm that it is of an acceptable scientific standard.

# Peer review: Reviewer response for version 1

**DOI:** 10.5256/f1000research.78730.r101953

**Published:** 2021-12-13

**Authors:** Nida Zahid

**Affiliations:** 1 Department of Surgery, Aga Khan University Hospital, Karachi, Pakistan

Summary:

 It is a study protocol of a systematic review to assess the risk factors for premature coronary artery disease (PCAD) in adults. Understanding modifiable and non-modifiable risk factors is critical for preventing morbidity and mortality from PCAD. All major risk factors, both modifiable and non-modifiable, have not been thoroughly investigated in previous systematic reviews among patients with PCAD. They either included specific risk factors for PCAD, targeted specific subgroups, or were limited to a single country. There has been no comprehensive appraisal of the evidence to date, despite the fact that there have been a number of published observational studies on PCAD risk factors, a comprehensive systematic review of the evidence is lacking. The findings of this systematic review will contribute to existing scientific knowledge and inform current clinical practice guidelines.

 Comments: 

 Data Synthesis:

 1. The authors have mentioned that for dichotomous variables odds ratio will be reported, however odds ratio may not be applicable for cohort studies and cross sectional studies for which the appropriate measures of association are relative risk and prevalence ratio respectively.

 2. The authors have mentioned in the title that it will be a systematic review, however in the methodology they have mentioned they will perform meta-analysis. Please make it consistent with the title.

Is the study design appropriate for the research question?

Yes

Is the rationale for, and objectives of, the study clearly described?

Yes

Are sufficient details of the methods provided to allow replication by others?

Yes

Are the datasets clearly presented in a useable and accessible format?

Not applicable

Reviewer Expertise:

NCDS, Mental health, Cancer, Male infertility

I confirm that I have read this submission and believe that I have an appropriate level of expertise to confirm that it is of an acceptable scientific standard.

# Response

**Published:** 2021-12-14

**Authors:** Adeel Khoja

**Affiliations:** University of Adelaide, Australia

Thanks so much for reviewing our systematic review protocol and providing us with feedback/comments.

 As per the first comment by the reviewer:

 1. ***The authors have mentioned that for dichotomous variables odds ratio will be reported, however odds ratio may not be applicable for cohort studies and cross sectional studies for which the appropriate measures of association are relative risk and prevalence ratio respectively.***

 Response: Thanks for this comment. We totally agree with the reviewer's comment. We will go as per the manner in which the studies have reported raw (crude) data and based on the two objectives of this systematic review, we assume the majority of the studies to be case-control in nature and hence we will report odds ratio. However, if we have cohort and cross-sectional studies as part of our systematic review, we will report relative risk and prevalence ratio accordingly.

 2. ***The authors have mentioned in the title that it will be a systematic review, however in the methodology they have mentioned they will perform meta-analysis. Please make it consistent with the title.***

 Response: Thanks for this comment. Since this is a protocol and we have planned for meta-analysis but this is not definitive at this stage. We will perform meta-analysis after looking at the number of studies for the two objectives outlined in the protocol.
